# Advances in Bone Joint Imaging-Metal Artifact Reduction

**DOI:** 10.3390/diagnostics12123079

**Published:** 2022-12-07

**Authors:** Sho Kohyama, Yuichi Yoshii, Yoshikazu Okamoto, Takahito Nakajima

**Affiliations:** 1 Department of Orthopaedic Surgery, Kikkoman General Hospital, Noda 278-0005, Chiba, Japan; 2 Department of Orthopaedic Surgery, Tokyo Medical University Ibaraki Medical Center, Ami 300-0395, Ibaraki, Japan; 3 Department of Diagnostic and Interventional Radiology, Faculty of Medicine, University of Tsukuba, Tsukuba 305-8575, Ibaraki, Japan

**Keywords:** digital tomosynthesis, computed tomography, magnetic resonance imaging, metal artifact reduction

## Abstract

Numerous types of metal implants have been introduced in orthopedic surgery and are used in everyday practice. To precisely evaluate the postoperative condition of arthroplasty or trauma surgery, periprosthetic infection, and the loosening of implants, it is important to reduce artifacts induced by metal implants. In this review, we focused on technical advances in metal artifact reduction using digital tomosynthesis, computed tomography, and magnetic resonance imaging. We discussed new developments in diagnostic imaging methods and the continuous introduction of novel technologies to reduce metal artifacts; however, these innovations have not yet completely removed metal artifacts. Different algorithms need to be selected depending on the size, shape, material and implanted body parts of an implant. Future advances in metal artifact reduction algorithms and techniques and the development of new sequences may enable further reductions in metal artifacts even on original images taken previously. Moreover, the combination of different imaging modalities may contribute to further reductions in metal artifacts. Clinicians must constantly update their knowledge and work closely with radiologists to select the best diagnostic imaging method for each metal implant.

## 1. Introduction

Advances in bone joint imaging in the past few decades have contributed to accurate diagnoses and the selection of appropriate treatments, particularly in the field of orthopedic surgery. Since many types of metal implants have been used in orthopedic surgery, reductions in metal artifacts are highly favorable for assessing the postoperative condition of arthroplasty or trauma surgery, periprosthetic infection, and the loosening of implants. In this review, we focus on technical advances in metal artifact reduction, which is essential in the orthopedic field to allow for precise evaluations of the conditions around metal implants. The purpose of this review was to discuss recent advances in metal artifact reduction in diagnostic imaging for orthopedic surgery as well as future perspectives.

## 2. Materials and Methods

The modalities we discuss are digital tomosynthesis (DT), computed tomography (CT), and magnetic resonance imaging (MRI). We searched for articles using Google scholar in the period of 2016–2022. Articles on DT were searched for using the keywords “digital tomosynthesis; metal artifact reduction; musculoskeletal”, those on CT using “CT; dual-energy CT; metal artifact reduction; musculoskeletal”, and those on MRI using “MRI; metal artifact reduction; musculoskeletal; sensing; sequences”. There were 269, 357, and 2250 articles, respectively, for each modality. We excluded case reports and articles that did not describe the method for metal artifact reduction and reviewed those with specific methods and related to clinical practice. In the first step, systematic reviews of each modality were examined for additional relevant articles. In the second step, we evaluated the original articles listed in these reviews and introduced established methods and their reduction of metal artifacts. 

## 3. Metal Artifact Reduction Strategies

### 3.1. Digital Tomosynthesis

A plain radiograph presents no metal artifacts and achieves high-density resolution, which allows us to assess different tissue types. Since all structures overlap each other on radiographs, it is difficult to identify structures in different layers with various depths. In other words, the depth resolution of radiography is low [1]. On the other hand, computed tomography (CT) has higher depth resolution, but with heavier metal artifacts. Furthermore, radiation exposure is almost ten-fold higher with CT than with radiography [2,3,4]. Traditionally, metal artifacts strongly and negatively affected the ability of CT to assess the fixation stability of implants, such as spot welds and radiolucent lines in the interface between bone and metal [5,6]. 

Digital tomosynthesis (DT) provides a set of section planes with a series of radiographic images obtained at various angles of a given anatomical region in a single sweep [7]. It has evolved since the beginning of the 2000s along with the use of digital flat panel detectors (FPD) [7]. Digital FPD improve image quality, reduce radiation exposure, and improve the productivity of radiography. DT has been applied to the clinical diagnosis of breast cancer or the identification of lung nodules [7,8,9,10]. It was recently introduced in the field of orthopedic surgery and is increasingly used to evaluate bone formation [7].

**Figure 1:**
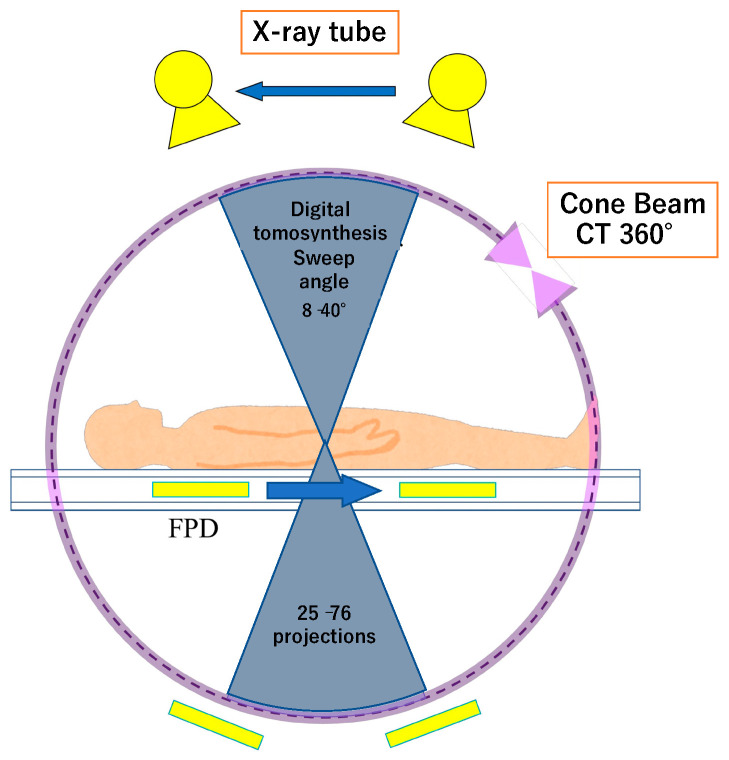
Basic principle of digital tomosynthesis (DT). DT acquires image data by a single sweep of an X-ray tube, with a range of 8 to 40°, whereas that of cone beam computed tomography is 360°. The X-ray is detected by digital flat panel detectors (FPD), which are either in the same position or may move in the opposite direction to the X-ray tube depending on the tables.

The principles of DT are as follows. The X-ray tube performs a sweep, with a range of 8 to 40°, which provides between 25 and 76 projections. While the X-ray tube is on sweep, FPD are either in the same position or may move in the opposite direction to the X-ray tube, depending on the tables. Regarding DT, independent acquisition parameters are the sweep angle, which is symmetrical, the sweep direction, the depth of the region of interest, the acquisition time, which varies between 2.5 and 12 s, and the number of projections [7,11]. Patients may be placed in various positions, which makes it possible to obtain images at arbitrary angles in addition to conventional acquisition in the coronal and sagittal planes [7] (Figure 1).

**Figure 2:**
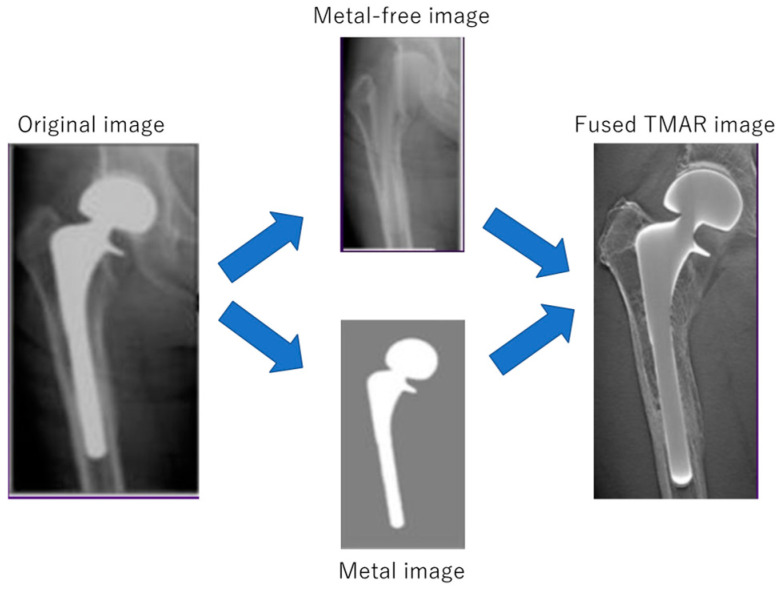
Brief image of tomosynthesis with metal artifact reduction (TMAR). Projection images were initially separated into metal and metal-free images. These images were iteratively reconstructed to reduce metal artifacts and were then fused to create TMAR images.

The important reconstruction algorithm for DT is an iterative reconstruction (IR) [7] because it is often used with a process called tomosynthesis with metal artifact reduction (TMAR), which is introduced using metal extraction and ordered subset-expectation maximization reconstruction [12]. This algorithm improves image quality and in-depth spatial resolution. Projection images were initially separated into metal and metal-free images, and iteratively reconstructed to reduce metal artifacts; these two images were then fused [5] (Figure 2). TMAR processing is better than radiography at reducing overlapping structures, with markedly lower radiation exposure doses and fewer artifacts than those with CT [13,14]. 

One of the major advantages of DT is its high in-section spatial resolution. The TMAR algorithm also improves depth resolution when metal objects are present. However, DT does not allow the production of multiplanar reconstructions, as in CT, due to the limited number of projections and the acquisition angle [7]. 

Even though DT reduces metal artifacts, some new artifacts are generated, such as undershooting, blurring-ripple, and ghost artifacts [7]. TMAR reduces these artifacts and improves the quality of the obtained image and in-depth spatial resolution [13,15], while simultaneously increasing the signal of metal objects in its peripheral part [7]. Inversion of the gray scale may improve the reading comfort of the tomosynthesis images; however, its diagnostic value remains unchanged [16].

In the diagnosis of fractures, DT is considered to be superior to radiography, but inferior to CT [7,17,18,19]. Its performance is optimal when the imaging plane is best suited to the analysis of target structures, such as the femoral neck and tibial plateau [7]. Tang et al. compared the sensitivity and specificity of DT with TMAR against radiography and conventional CT for detecting implant loosening in total hip arthroplasty (THA), namely, radiolucent lines narrower than 2 mm surrounding cementless femoral stems, in four cadavers [20]. The overall sensitivities of DT, radiography, and CT were 63.3, 20.5, and 50.2%, respectively. The sensitivity of DT was significantly higher than those of the other two modalities. They concluded that DT has the potential to increase the diagnostic accuracy of early prosthetic loosening after cementless THA in clinical practice. 

In a cohort study of 48 patients, Tang et al. reported that the diagnostic accuracy of DT with TMAR to assess the fixation stability of cementless THA was significantly higher than those of plain radiography and CT for both the femoral and acetabular sides [1]. They assessed obtained images with following diagnostic criteria; loosening, not loosening (definite osteointegration fixation), and possible loosening (not sure). They confirmed fixation stability with two major clinical criteria as a reference standard, intraoperative mechanical tests and postoperative retrieval findings. They compared image findings and clinical standards, and categorized them into ‘accurate’, ‘wrong’ and ‘not sure’ groups. They defined diagnostic accuracy as the rate of the ‘accurate’ group for each imaging examination. Diagnostic accuracies for the femoral stem and acetabular cup were 82.6 and 84.5%, 44.6 and 67.3%, and 39.6 and 74.6% with DT, radiography, and CT, respectively. They concluded that DT with TMAR increased the diagnostic accuracy of assessments of the fixation stability of cementless THA by minimizing metal artifacts in the border between the implant and bone and clearly depicting peri-implant trabecular structures. Guo et al. reported similar findings for the usefulness of DT with TMAR in evaluations of periprosthetic conditions [21]. They investigated the osteointegration of 24 patients who underwent revision cementless THA. All the patients underwent radiography, DT with TMAR, and CT prior to the surgery. Evidence of osteointegration in retrieved prostheses was used as the reference standard. They evaluated 13 femoral stems and 14 acetabular components. Sensitivities on the femoral side for radiography, DT with TMAR and CT were 50.4%, 73.8% and 36.4%, respectively. Sensitivities on the acetabular side for radiography, DT with TMAR and CT were 45.9%, 60.2% and 45.1%, respectively. Accordingly, specificities on the femoral sides were 87.8%, 94.3%, and 90.9%, and on the acetabular sides were 66.4%, 86.4% and 73.5%, respectively.

On the other hand, Gillet et al. demonstrated that the diagnostic performance of DT in hip prosthetic loosening was similar to that of radiographs, and its sensitivity was lower than that of CT with the metal artifact reduction algorithm (MAR) [22]. They investigated 49 patients with painful hip prostheses. Among them, 21 cases were confirmed as prosthetic loosening by surgery. Sensitivities for radiography, DT and CT with MAR were 33.3–51.5%, 39.9–45.4%, and 84.5%, respectively. Specificities were 96.9–100%, 98.5–100%, and 95.4–96.9%, respectively. Since the interobserver agreement of DT was higher than that of radiography (0.53 vs. 0.39), particularly for the acetabular component, they recommended the use of DT in combination with conventional radiography. In cases with an inconclusive initial evaluation with radiography and digital tomosynthesis, additional evaluations by CT with MAR were recommended [22].

Metal artifact reduction by DT is advantageous for assessing bone formation after various surgical procedures other than arthroplasties, particularly in the presence of metal implants. Toyooka et al. investigated 27 patients and reported that the evaluation of bone integration after anterior cruciate ligament reconstruction of the knee with DT was equivalent to that with CT within 15% of diagnostic error. DT showed a sensitivity of 79–96%, specificity of 64–100% and diagnostic accuracy of 81–96% [23]. Ishibashi et al. demonstrated the applicability of DT to assessments of postoperative bone formation in the opening gap after open wedge high tibial osteotomy (OWHTO). They investigated the correlation between gap filling value (GFV) and modified van Hemert’s score (MVHS)for assessment of bone formation after OWHTO. GFV and MVHS showed a strong correlation (r = 0.630, *p* < 0.001). The interclass correlation coefficient (ICC) for intraobserver reliability was 0.958 for GFV and 0.978 for MVHS. ICC for interobserver reliability was 0.975 for GFV and 0.950 for MVHS. They concluded that the evaluation of bone formation after OWHTO using DT has high accuracy and reproducibility [24]. 

**Figure 3:**
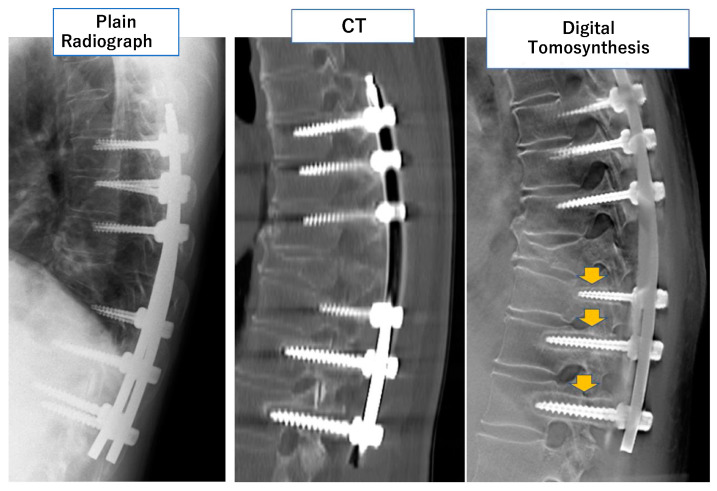
Comparison among plain radiography, computed tomography (CT), and digital tomosynthesis (DT) in an evaluation of pedicle screw loosening after spinal instrumentation. In DT, radiolucent areas around PS were more evident than in images obtained by the two other modalities (arrows).

DT is also useful in the field of spine surgery. Okano et al. developed a novel pixel selection method for pedicle screws (PS) by DT [25]. They enlarged the obtained images up to 3200 times and observed the luminance of each pixel to identify the peripheral line of the PS. Using the identified peripheral pixels, they identified rod curve lines. From the (x, y) coordinates of ten pixels on the rod curve line, they calculated quadratic regression curves. This method is useful when identifying the same cross-sectional slice from two different datasets obtained separately. The two slices are on the same cross-section if the shapes of the regression curves are the same. Using this method, Mataki et al. evaluate PS loosening in 41 patients with 72 PS by DT. They concluded that DT has the potential to diagnose PS loosening more accurately and quantitatively than conventional modalities [26] (Figure 3).

The most recent advance in DT is the application of a deep learning program to achieve more advanced metal artifact reduction. Gomi et al. reported a novel projection-based cross-domain learning framework for MAR [27]. They used the novel algorithm and successfully reduced metal artifacts more than with conventional TMAR algorithms. They also succeeded in reducing the radiation dose by 55% [27]. 

In summary, DT provides higher in-section spatial resolution than plain radiography, with markedly fewer metal artifacts and lower radiation doses than with conventional CT. Even though multiplanar reconstruction is impossible, DT is a useful tool for evaluating periprosthetic conditions after arthroplasty and bone formation after fractures or osteotomies. In cases in which the assessment of soft tissues is not needed and the lesion of interest is limited, DT may outperform CT or MRI after arthrography [15,28] (Table 1). 

### 3.2. Computed Tomography

Computed tomography (CT) is commonly used to evaluate complications related to metal implants, such as infection, loosening, fracture, implant failure, particle disease, tumors, and pseudotumors [29]. However, a combination of metal artifacts is a major limitation in evaluations by conventional CT. Examples of artifacts are beam hardening, scattering, photon starvation, and edge effects [5,6,30,31]. These metal artifacts result in the reconstruction of near-metal tissue by corrupted data [31]. The severity of metal artifacts on CT is dependent on the atomic number as well as the shape and size of metal implants. Larger implants and metal with higher atomic numbers result in greater metal artifacts [32].

There are three main strategies to achieve metal artifact reduction: modifying standard acquisition and reconstruction, modifying projection data and/or image data, and applying dual-energy CT (DECT) [31]. Increases in the tube voltage (kV) and tube current (mAs) are traditional techniques to reduce beam hardening and photon starvation in conventional CT [31,33]. A reduction in total collimation (detector width) decreases scatter effects [31]. Instead of using standard filtered back-projection (FBP), changing the reconstruction algorithm to projection interpolation techniques or more advanced IR and model-based iterative reconstruction (MBIR) algorithms reduces the scatter and edge effects [29,30,31,34]. However, these techniques require a higher radiation dose and may result in lower spatial and contrast resolution as well as reconstruction errors [35]. 

**Figure 4:**
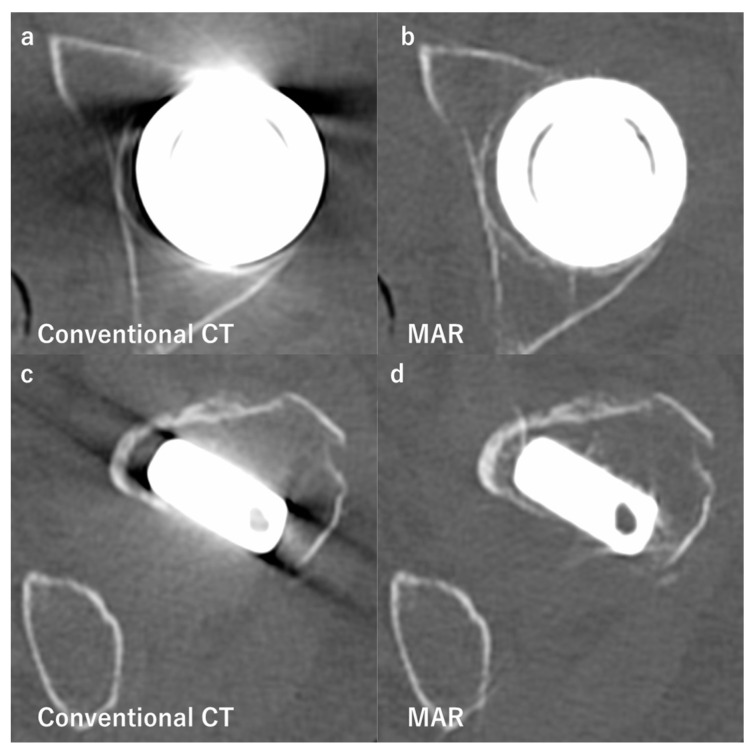
Comparison of conventional computed tomography (CT) and images reconstructed with the metal artifact reduction algorithm (MAR). Axial images of a patient after hemiarthroplasty of the hip. Images (**a**,**b**) show the bipolar femoral head, while (**c**,**d**) show the femoral stem at the level of the minor trochanter. Images (**a**,**c**) were obtained by conventional CT, and show strong beam hardening, scattering, photon starvation, and edge effects. On the other hand, there are no obvious metal artifacts on images (**b**,**d**) using MAR. It leads to better visualization of the outlines of the prosthesis.

The modification of projection and/or image data includes the use of the metal artifact reduction algorithm (MAR), which reconstructs more accurate images by segmenting and extracting metal artifacts iteratively [31]. MAR significantly reduces metal artifacts, particularly the effects of beam hardening and photon starvation [31,32] (Figure 4). The use of MAR in addition to MBIR was previously reported to reduce the radiation dose by up to 80% from that with conventional FBP [31]. Previous studies demonstrated the effectiveness of MAR at reducing metal artifacts following total hip arthroplasty (THA) [36,37,38,39], total knee arthroplasty (TKA) [30], and different metal fixation implants [40,41,42]. Bolstad et al. concluded that MAR reduced metal artifacts, particularly on implants made of steel and cobalt-chrome [42]. However, it is important to note that the use of MAR may induce new minor artifacts in the tissues inside and proximal to metal objects that are not visible on conventional CT, such as streaks or the disappearance of metal implants. The inaccurate segmentation of data or errors in the estimation of corrupted data has been shown to cause these artifacts [31,42,43].

Tissues and materials have different attenuation properties at different energy levels, and the difference in density between a metal implant and musculoskeletal tissue is the main cause of a metal artifact [30]. Soft tissue, water, and adipose tissue show consistent attenuation levels throughout any X-ray beam energy. However, the attenuation levels for calcium and iodine are higher for a lower beam energy level due to the photoelectric effect [44]. DECT takes advantage of this by acquiring CT attenuation data at two different energy levels, generally 70–80 and 140–150 kV [45,46]. After data acquisition, virtual monoenergetic images (VMI) are reconstructed, which is the main characteristic and advantage of DECT. VMI are reconstructed gray-scale images that virtually simulate appearances, which may be achieved using a true monochromatic X-ray beam [33]. 

VMI are useful because they are reconstructed at arbitrary average energy levels and, thus, contrast optimization and artifact reduction are possible. By making VMI with higher virtual monochromatic energies, the influence of low-energy photons may be reduced, leading to fewer beam-hardening artifacts without increasing the radiation dose [31,47,48,49]. However, DECT cannot reduce photon starvation, scatter, or edge effects.

There are three main types of postprocessing algorithms for DECT datasets: image optimization algorithms (IOA), differentiation algorithms (DA), and quantification algorithms (QA) [45,46]. IOA typically provides two sets of monochromatic images and a nonlinear blended image of high- and low-energy images. Monochromatic images are obtained at 80 or 100 kV and 140 kV. Low-energy images generally provide high contrast, while high-energy images provide low noise. In DA, specific materials may be subtracted from the data, or two materials may be differentiated by color coding. MAR is considered to be one of the differentiation algorithms. QA is based on the decomposition of three materials and provides color-coded images of certain material in postcontrast examinations [44].

Previous studies reported that DECT reduced metal artifacts on spine implants, implants for fractures, and hip and knee prostheses [40,41,42,50,51,52]. However, there is no generalized optimal kV for metal implants, which may be attributed to differences in metal alloys, the size, shape, and geometry of the implant, the body region, and the acquisition parameters used [31]. However, most studies reported a range of 110–150 kV, with 130 kV being sufficient for most small implants composed of lightweight alloys [31,50,52,53]. 

Lee et al. compared artifact reduction and image quality between DECT and conventional CT. They investigated monochromatic extrapolation at 70 and 150 keV in 40 patients with metal implants and performed conventional CT on 40 matched controls with metal implants [54]. The high kV reconstruction of DECT showed significantly higher values within muscle (−96 HU vs. −405 HU) and fat tissues (−115 HU vs. −301 HU) surrounding the implant, significantly lower mean image noise (75 HU vs. 129 HU), a higher signal-to-noise ratio (−0.8 vs. −4.3), and superior image quality over conventional CT. Furthermore, mean radiation doses were similar between DECT and conventional CT (14.2 mGy vs. 19.3 mGy) [54].

Donders et al. investigated 41 patients with suspected non-union after fracture surgery using intramedullary nails and plates [55]. They performed DECT at high (130–150) kV and low (70) kV and used Likert scores to evaluate the usefulness. It was concluded that the image quality (1.83 vs. 0.88) and diagnostic confidence were higher (2.37 vs. 1.43) and the false negative rates of non-union were 5% lower with high kV DECT than with low kV DECT. Non-union was confirmed during revision surgery [55]. These findings support the effectiveness of metal artifact reduction by DECT in actual practice.

Other than metal artifact reduction, DECT has been used to detect urate crystals in gout or calcium pyrophosphate dehydrate crystal arthropathy as well as bone marrow edema in trauma or inflammation and also to characterize tendons, ligaments, and intervertebral discs [46]. However, VMI may decrease tissue contrast because low-energy photons increase contrast [32]. In addition, when implants are large and have sharp edges or high-molecular-weight metals, DECT artifact reduction is less effective [45]. 

Barreto et al. compared the effectiveness of MAR, conventional CT images, and images obtained from six cadavers containing metal implants in the head, neck, abdomen, pelvis, and extremities using DECT [43]. Left hip bipolar hemiarthroplasty, TKA, and an implant for anterior cervical disc fusion were included as musculoskeletal implants. The severity of metal artifacts, the visualization of anatomical structures, and the assessors’ confidence in diagnostic interpretation were assessed using the original 5 points scale. In all six cadavers, MAR was preferred over conventional CT images, while conventional CT images were preferred over DECT images. Since DECT reduced soft tissue contrast and streak artifacts remained, it was difficult to evaluate the surrounding structures. The cervical spine was the only case in which DECT reduced the severity of metal artifacts and increased the visibility of all structures. They concluded that MAR was more effective at reducing metal artifacts than DECT; however, new minor streak artifacts were observed in MAR images. When the anatomy or implant is relatively small, DECT may be superior to MAR without additional artifacts [43].

**Figure 5:**
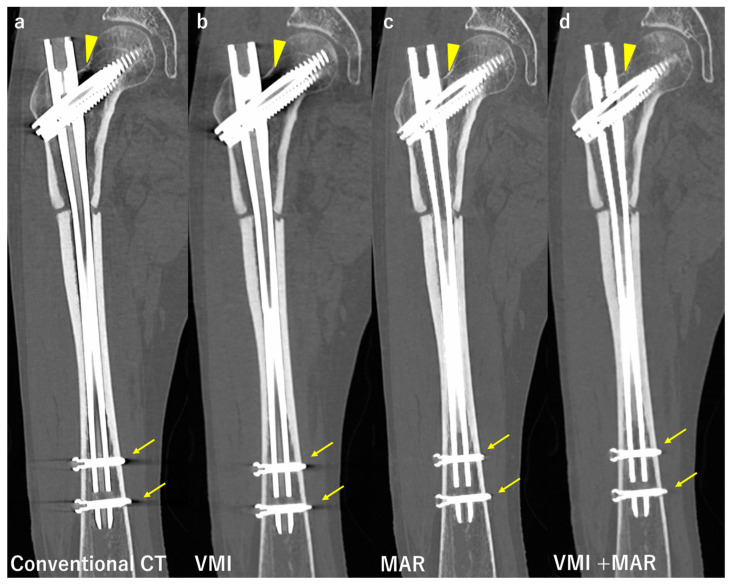
Comparison of conventional computed tomography (CT) and images reconstructed with virtual monoenergetic images (VMI), the metal artifact reduction algorithm (MAR), and combination of VMI and MAR. Coronal images of a patient after intramedullary nail fixation of the femur. Image (**a**) was taken by conventional CT at 120 kV, and shows strong beam hardening, scattering, photon starvation, and edge effects at the junction of the nail and lag screw (arrowheads), and at the tip of distal locking screws (arrows). Image (**b**) was taken by VMI at 135 kV, and the artifacts are slightly reduced, especially at the tip of distal locking screws. Image (**c**) was taken with MAR, and the artifact reduction is stronger than VMI, only very small dark streaks both at the junction of the nail and lag screw and at the tip of distal locking screws. Image (**d**) was taken in combination with VMI at 135 kV and MAR, and there are almost no artifacts present, leading to a better visualization of the outlines of the prosthesis.

Previous studies suggested that VMI by DECT in combination with advanced reconstruction algorithms, such as projection interpolation techniques and MAR, were more likely than VMI alone to reduce beam hardening artifacts [45]. Neuhaus et al. reported that the application of VMI or MAR was not sufficient to reduce severe metal artifacts induced by monolateral or bilateral hip prostheses. They concluded that the combination of VMI and MAR achieved greater reductions in metal artifacts. Additionally, the assessment of adjacent structures such as pelvic organs and bone improved the most with the combination of VMI and MAR [41]. Andersson et al. investigated several MAR and VMI for the metal artifact reduction of bilateral hip prosthesis phantom and showed that the use of VMI alone did not decrease the artifact to the same extent as the combination of VMI and MAR [56]. Furthermore, in the region of interest between bilateral hip prostheses, the artifact even increased with the use of VMI. Bongers et al. compared the metal artifact reduction performance of blended polychromatic images (equivalent to 120 kV) and monochromatic images at 130 kV with and without MAR on 20 patients with hip prostheses and 30 patients with dental implants [57]. They evaluated the artifact reduction rate qualitatively and quantitatively. Compared to conventional CT, DECT reduced by 33% and 8%, MAR reduced by 56% and 71%, and a combination of DECT and MAR reduced by 76% and 76% for hip prosthesis and dental implants, respectively. Long et al. examined twenty patients with instrumented spines using VMI, MAR, and their combination [58]. They used original artifact scores (1 to 5 points) and image quality scores (1 to 4 points), and VMI in combination with MAR showed the best for both scores. ICC was 0.779 for the bony structures and 0.892 for the soft tissues. Bongers et al. and Long et al. both concluded that the combination of VMI and MAR achieved the greatest reductions in metal artifacts (Figure 5).

Yue et al. evaluated 35 patients with unilateral hip arthroplasty using high (120–140) and low (80–100) kV VMI with and without MAR. Their findings showed that metal artifacts were significantly lower in high kV VMI with MAR, and the image quality of the peri-prosthesis region was better [59]. Chae et al. assessed 57 knees from 36 patients after TKA. They compared images obtained by conventional CT, MAR, VMI, and the combination of MAR and VMI, and found that the combination of MAR and VMI achieved the greatest reductions in metal artifacts and more accurately depicted soft tissues. On the other hand, bony structures were more accurately depicted by MAR alone [60]. These findings suggest the usefulness of the combination of VMI and MAR in evaluations of peri-prosthesis conditions at both the hip and knee.

Similar to DT, deep learning is recently being used in MAR in CT. Park et al. employed U-Net to reduce artifacts in polychromatic CT against beam hardening [61,62]. Zhang et al. concluded that a convolutional neural network was capable of creating priority images with fewer artifacts to correct the regions of a sinogram corrupted by metal artifacts [63]. In combination with MAR, these methods achieved effective metal artifact reduction. However, the processing of new artifacts in reconstructed CT images is still challenging [27]. Recent studies suggested that the use of a generative adversarial network increased the recognition and tissue segmentation accuracy of tissues on CT images [27,64]. In combination with MAR, it may effectively reduce metal artifacts. Future advances in the field of deep learning are expected to further reduce metal artifacts on medical images. 

In summary, various approaches are employed to reduce metal artifacts on CT. Based on the findings described above, the combination of DECT and MAR is currently the best solution for metal artifact reduction. However, since MAR may create new artifacts in the case of lightweight metals, this combination may fail to effectively reduce metal artifacts [31]. Therefore, to adjust the metal artifact reduction approach, minimize artifacts, and optimize the image quality and diagnostic value of CT, it is important for clinicians to provide implant-specific information to radiologists because metal artifacts differ due to differences in the size, geometry, and alloys of metal implants [31] (Table 2). 

### 3.3. Magnetic Resonance Imaging

**Figure 6:**
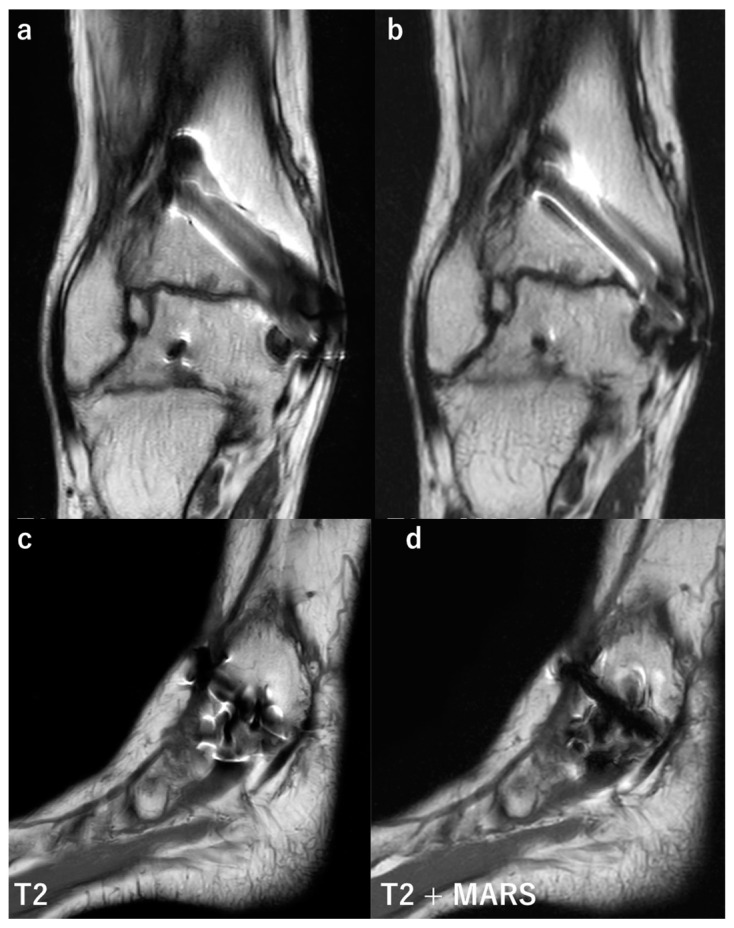
Comparison of conventional MRI and images obtained with MARS. Coronal and sagittal MR images of a patient after medial malleolus fracture of the right ankle. The fracture was treated by a screw fixation. The coronal MR image (**a**) was taken by conventional T2 weighted image (T2WI), and significant signal loss and distortion around the implant are present. Image (**b**) was taken by T2WI with MARS, showing a better visualization of tissue around the screw. The sagittal image (**c**,**d**) was taken by T2WI, and T2WI with MARS, respectively. Similarly, with coronal images, signal loss and distortion are reduced with MARS, leading to better visualization of tissue surrounding the implant.

Magnetic resonance imaging (MRI) plays an important role in the assessment of musculoskeletal disorders, including bone and soft tissues. However, due to heavy metal artifacts, MRI on a patient with metal implants leads to poor image quality and, thus, has been avoided [65]. Over the last few decades, various sequences have been introduced to obtain ideal images with fewer metal artifacts (Figure 6). The metal artifact reduction sequence (MARS) was described by Olsen in 2000 [66]. Recent studies reported the usefulness of MARS for diagnosing periprosthetic infections of the shoulder [67] and hip and knee [68]. More advanced sequences for metal artifact reduction have been introduced, such as the view angle tilting (VAT) technique [69], WARP (a sequence commercialized by Siemens Healthcare, Erlangen, Germany) [65], slice encoding for metal artifact correction (SEMAC) [70], multi-acquisition variable-resonance image combination (MAVRIC) [71], and MAVRIC selective (MAVRIC SL) [72].

VAT is a strategy that is used to decrease in-plane artifacts. It reduces signal loss and pile-up artifacts by adding an altered readout gradient [69]. However, VAT causes a small degree of blurring and cannot correct through-section distortions [65]. WARP is intended to optimize the standard principles of MARS and includes multidirectional VAT to achieve further reductions in in-plane distortions [65]. SEMAC applies additional phase-encoding gradients perpendicular to the slice (*z*-direction) prior to signal readout in order to correct through-plane distortions, such as non-planar slice excitation [70,71]. It corrects metal artifacts by the strong encoding of each excited slice against metal-induced field inhomogeneities, which addresses both in-plane and through-plane distortions [71]. VAT and SEMAC were reported to be useful in evaluations of patients after THA and TKA [73,74]. 

Galley et al. examined 40 patients with periprosthetic infections after THA using 1.5 T MRI based on the coronal short inversion time inversion-recovery (STIR)-SEMAC sequence. The detection of a periosteal reaction, capsule edema, and intramuscular edema in evaluations of periprosthetic joint infections had sensitivities of 78, 83, and 95%, specificities of 90, 95, and 86%, and accuracies of 86, 91, and 89%, respectively. Interobserver agreement was almost perfect, with ICC values between 0.88 and 0.92 [75]. Takahashi et al. investigated the reliability of SEMAC to detect prosthesis loosening by comparing it to surgical outcomes in 47 patients after THA on 55 hips. Patients were divided into two groups, those with a painful hip (group P) and those without pain (group C). MRI was scored for osteolysis and bone marrow edema. Eleven patients in group P required revision surgery, in contrast to 0 in group C. Correlations between SEMAC and revision surgery outcomes were moderate to weak (Γ = 0.415 on T1W-SEMAC, and Γ = 0.35 on STIR-SEMAC). Sensitivity, specificity, positive predictive value, and negative predictive value (NPV) in group P were 72.7, 64.3, 44.4, and 85.7%, respectively, with T1W-SEMAC, 90.9, 46.4, 40.0, and 92.9%, respectively, with STIR-SEMAC, and 36.3, 78.5, 40.0, and 75.8%, respectively, with proton density-weighted (PDW)-SEMAC. They concluded that STIR-SEMAC achieved high sensitivity and NPV for detecting periprosthetic fluid and marrow edema, indicating prosthetic loosening [76]. 

Jungmann et al. compared the combination of VAT and SEMAC with conventional MRI to assess its usefulness in evaluations of large orthopedic tumor endoprosthesis in 25 patients. They used a five-point scale to assess qualitative parameters. Artifact diameters and distortions were significantly reduced in the VAT and SEMAC groups. Two cases of tumor recurrence, ten of infection, and thirteen other pathologies were diagnosed. They suggested that the combination of VAT and SEMAC was beneficial for detecting periprosthetic pathologies in postoperative follow-ups due to significant reductions in metal artifacts [69].

MAVRIC is a spin-echo-based sequence that minimizes image distortions by combining multiple individual datasets acquired at frequency bands that are gradually offset from the dominant proton frequency. It is capable of reducing both through-section and in-plane artifacts [65,71]. MAVRIC SL has the advantages of both STIR and MAVRIC. STIR allows for more uniform fat suppression around metals than spectral fat saturation. MAVRIC SL maintains metal artifact corrections and the high signal-to-noise ratio (SNR) of MAVRIC using an overlapped spectral strategy with multiple frequency-selective excitations [72]. Zochowski et al. conducted a feasibility study to compare the quality and diagnostic utility of three metal artifact reduction sequences in evaluations of 84 patients after THA. The investigated sequences were MAVRIC SL, isotropic MAVRIC SL, and reduced repetition time isotropic MAVRIC SL using a 1.5T imager. In comparisons with conventional MAVRIC SL acquisitions, they concluded that isotropic MAVRIC SL acquisitions improved SNR, the findings of lesions, such as the presence of low-signal-intensity deposits, osteolysis, the loosening of a prosthesis, and the visualization of synovium and periprosthetic bone, and reduced blurring. Interrater and intrarater agreement were substantial to almost perfect for the clinical features investigated (ICC values 0.61–1.00). However, overall SNR and visualization of synovium on conventional MAVRIC SL images tend to have a low agreement, with an ICC value of 0.26 and 0.08, respectively [77]. Kim et al. conducted a preliminary study to investigate the usefulness of MAVRIC SL STIR in evaluations of the postoperative cervical spine with artificial disk replacement among five volunteers and a cadaver using a 3T imager. The images obtained with MAVRIC SL STIR were compared with STIR images. MAVRIC SL STIR showed fewer signal void areas and distortions and better visualization of anatomical structures. However, the image quality of the spinal cord was better with STIR. For the cadaveric study, interobserver agreement was substantial (κ = 0.7). For the volunteer study, the agreement was almost perfect (κ = 0.89). They concluded that MAVRIC SL STIR may be useful in the evaluations of surgical sites after artificial disk replacement by signal void reductions and distortions [72]. 

As imaging sequences become more complex, imaging times have increased. Recent advances in acceleration techniques allow for more precise and rapid examinations. Parallel imaging (PI), simultaneous multi-slice acquisition, compressed sensing (CS)-based sampling, and synthetic MRI techniques are acceleration techniques that shorten acquisition times in a linear manner. CS acceleration is ideally suited for SEMAC and MAVRIC due to its high intrinsic sparsity [78].

PI acceleration reduced the acquisition times of axial, sagittal, and coronal T1W and STIR SEMAC pulse sequences to clinically feasible acquisition times of 5–6 min. With the combination of CS acceleration and elliptical scanning, SEMAC acquisition times may be reduced by 60–70% to 4–5 min per sequence [79,80]. Previous studies reported the clinical application of eight-fold CS-accelerated STIR, T1W, PDW, and T2W SEMAC pulse sequences for metal artifact reduction in MRI examinations of patients with hip [75,81,82,83], knee [84], and ankle [85,86] arthroplasty implants, achieving 60–70% reductions in acquisition times from PI-accelerated protocols.

In summary, more advanced imaging sequences for metal artifact reduction on MRI have been introduced in recent years. The combination of the CS acceleration technique with SEMAC currently appears to be the best solution in MRI to achieve metal artifact reductions with a reasonable acquisition time [87]. An MR imager with a stronger magnetic field, such as 7-Tesla, has recently been used, and the advanced metal artifact reduction techniques described herein appear to be efficient [88]. It is important to remain up-to-date on advances in imaging techniques and select the most suitable criteria for each patient (Table 3).

## 4. Future Perspectives and Conclusions

The number of patients undergoing arthroplasty surgeries has increased worldwide to more than one million per year [1]. In addition to arthroplasty surgeries, surgeons use intramedullary nails and locking plates in patients with various fractures. Increases in the number of surgeries are associated with a higher number of complications, such as periprosthetic infections and fracture non-union. Since their rapid diagnosis and treatment are favorable, metal artifact reduction techniques are essential for a precise diagnosis. Accurate imaging is necessary for the appropriate decision-making regarding treatments. 

Advances have been achieved in diagnostic imaging technologies, and novel technologies are continuously being introduced. However, this review indicates that the latest technology has not yet been fully applied clinically. Clinicians must constantly update their knowledge and work closely with radiologists to select the best diagnostic imaging method for each case (Table 4). 

As reviewed herein, there are various approaches to reducing metal artifacts. Different algorithms need to be selected based on the size, shape, material and implanted body parts of the implant. Future advances in metal artifact reduction algorithms and techniques and the development of new sequences will enable further reductions in metal artifacts even on original images taken previously. In addition, the combination of different imaging modalities may contribute to further developments in the field of metal artifact reductions. In intraarticular lesion imaging, a novel technique for the three-dimensional fusion of MRI and CT has been introduced [89,90]. Each imaging technique may compensate for the other’s shortcomings and contribute to an accurate diagnosis by clinicians.

## Figures and Tables

**Table 1:** Overview of representative DT studies. Major results are presented. AUC value was not mentioned in the literature unless described. Digital tomosynthesis: DT; tomosynthesis with metal artifact reduction: TMAR; computed tomography: CT; total hip arthroplasty: THA; anterior cruciate ligament: ACL; interclass correlation coefficient: ICC.

	Compared Modalities	Subject	Results
Tang et al. [21]	Radiography, DT with TMAR, CT	4 cadaveric femurs Femoral stem	Sensitivity Radiography 20.5% DT with TMAR 63.3% CT 50.2% Specificity Radiography 92.5% DT with TMAR 87.5% CT 82.5%
Ottenin et al. [18]	Radiography, DT CT	100 patients with acute wrist trauma Carpal bones	Sensitivity Radiography 61–80% DT 77–87% CT 93–95% Specificity Radiography 65–83% DT 76–82% CT 86–95%
Tang et al. [1]	Radiography, DT with TMAR, CT	48 patients with cementless THA (Femoral stem and acetabular cup)	Diagnostic accuracy Femoral stem Radiography 84.5% DT with TMAR 82.6% CT 44.6% Acetabular cup Radiography 39.6% DT with TMAR 67.3% CT 74.6%
Guo et al. [22]	Radiography, DT with TMAR, CT	24 patients with cementless THA 13 femoral stems and 14 acetabular components were evaluated.	Sensitivity Femoral side Radiography 50.4% DT with TMAR 73.8% CT 36.4% Acetabular side Radiography 45.9% DT with TMAR 60.2% CT 45.1% Specificity Femoral side Radiography 87.8% DT with TMAR 94.3% CT 90.9% Acetabular side Radiography 66.4% DT with TMAR 86.4% CT 73.5%
Gillet et al. [23]	Radiography DT CT + MAR	49 patients with painful hip prostheses. Evaluated prosthestic loosening.	Sensitivity Radiography 33.3–51.5% DT 39.9–45.4% CT + MAR 84.5% Specificity Radiography 96.9–100% DT 98.5–100% CT + MAR 95.4–96.9%
Toyooka et al. [24]	DT CT	Bone integration of 27 patients who underwent ACL reconstruction was evaluated	DT was equivalent to CT for the evaluation of bone plug integration within a 15% diagnostic error. Sensitivity 79–96% Specificity 64–100% Diagnostic accuracy 81–96%
Ishibashi et al. [25]	DT	Open Wedge High Tibial osteotomy Gap filling value (GFV) and modified van Hemert’s score (MVHS)	GFV had strong correlation with MVHS (r = 0.630, *p* < 0.001) ICC value for intraobserver reliability GFV 0.958 MVHS 0.978 ICC value for interobserver reliability GFV 0.975 MVHS 0.950
Mataki et al. [27]	DT	Pedicle screw (PS) displacement angle Loosening group vs. group without PS loosening	The displacement angle was significantly greater in loosening group (5.7° vs. 0.6°) Sensitivity 100% Specificity 93% AUC = 0.98

**Table 2:** Overview of representative CT studies. Major results are presented. AUC value was not mentioned unless described. Computed tomography; CT, virtual monoenergetic images; VMI, metal artifact reduction algorithm; MAR, SNR; signal to noise ratio, total knee arthroplasty; TKA, total hip arthroplasty; THA, interclass correlation coefficient; ICC.

	Compared Modalities	Subjects	Results
Lee et al. [55]	Conventional CT VMI (70 and 150 kV)	40 patients with metallic implants	VMI at high kV reduced metal artifacts, increased SNR, and improved image quality.
Donders et al. [56]	VMI Low (70) kV versus high (130–150) kV	41 patients with a clinical suspected non-union with hardware in place. Likert scores were used.	Image quality 1.83 (high kV) > 0.88 (low kV) Number of false-negative non-unions; 5% reduced by high kV. Diagnostic confidence 2.37 (high kV) > 1.43 (low kV)
Barreto et al. [44]	Conventional CT, MAR, VMI	Cadavers with hip bipolar hemiarthroplasty, TKA, and an implant for anterior cervical disc fusion.	Rank of the original 5 points scale Hip; MAR > CT > VMI TKA; MAR > CT > VMI Spine; VMI > MAR > CT
Neuhaus et al. [42]	Conventional CT, MAR, VMI, VMI + MAR	24 patients after THA	VMI + MAR reduced artifacts the most. VMI + MAR improved the assessment of adjacent structures the most.
Andersson et al. [57]	Conventional CT, MAR, VMI, VMI + MAR	Bilateral hip prosthesis phantom	Artifact reduction rate MAR 52–75% VMI 12–52% (in a certain region artifact increased up to 32%) VMI + MAR 75–77%
Bongers et al. [58]	Conventional CT, MAR, VMI, VMI + MAR	Hip prosthesis and dental implants. Qualitative and quantitative evaluation.	Artifact reduction rate (Hip, dental implant, respectively) VMI 33%, 8% MAR 56%, 71% VMI + MAR 76%, 76%
Long et al. [59]	MAR VMI VMI + MAR	20 patients with instrumented spines. Artifact score (1 to 5) Image quality score (1 to 4)	VMI + MAR showed the best artifact and image quality scores. ICC 0.779
Yue et al. [60]	VMI VMI + MAR (80, 100, 120 and 140 kV)	35 patients with THA. Artifact index (AI) CT number Subjective scores	AI in VMI + MAR at 120 and 140 kV were significantly lower than others. Accuracy of CT numbers for the peroprosthetic region improved with VMI + MAR. VMI + MAR at 120 and 140 kV had higher subjective scores.
Chae et al. [61]	Conventional CT MAR VMI VMI + MAR	57 patients with TKA Area of the artifacts Mean attenuation Artifact index (AI) Contrast-to-noise ratio (CNR)	VMI + MAR showed the best performance in artifact reduction and soft tissue depiction. MAR depicted bony structures the best.

**Table 3:** Overview of representative MRI studies. Major results are presented. AUC value was not mentioned unless described. Magnetic resonance imaging: **MRI**; short inversion time inversion-recovery: **STIR**; proton density weighted: **PDW**; slice encoding for metal artifact correction: **SEMAC**; view angle tilting: **VAT**; multi-acquisition variable-resonance image combination: **MAVRIC**; MAVRIC selective: **MAVRIC SL**; repetition time: TR; interclass correlation coefficient: ICC; positive predictive value: PPV; negative predictive value: **NPV**; total hip arthroplasty: **THA**.

	Modalities, Sequences	Subjects	Results
Galley et al. [77]	1.5 T system STIR-SEMAC	40 patients with periprosthetic infections after THA Periosteal reaction, capsule edema, and intramuscular edema were evaluated.	Sensitivities 78, 83, 95%, respectively, Specificities 90, 95, 86%, respectively, Accuracies 86, 91, 89%, respectively, Interobserver agreement ICC values 0.88–0.92
Takahashi et al. [78]	1.5 T system T1WI-SEMAC STIR-SEMAC PDW-SEMAC	47 patients after THA Prosthesis loosening was evaluated.	T1WI-SEMAC Sensitivity 72.7% Specificity 64.3% PPV 44.4%, NPV 85.7% STIR-SEMAC Sensitivity 90.9%, Specificity 46.4%, PPV 40.0%, NPV 92.9% PDW-SEMAC Sensitivity 36.3% Specificity 78.5% PPV 40.0%, NPV 75.8%
Jungman et al. [71]	1.5T system Conventional MRI VAT VAT + SEMAC (STIR, T1W, T2W were taken for each group)	25 malignant bone tumor patients after surgery (metal implants used) with clinical suspicion of tumor recurrence.	VAT + SEMAC reduced artifact diameters and distortions (*p* < 0.001). VAT + SEMAC improved diagnostic confidence (*p* < 0.05). Two cases of tumor recurrence were diagnosed.
Zochowski et al. [79]	1.5T system Conventional MAVRIC SL Isotropic MAVRIC SL Reduced TR MAVRIC SL	84 patients after THA	Isotropic MAVRIC SL and reduced TR MAVRIC SL decreased blurring and improved visualization of the synovium and the periprosthetic bone (*p* < 0.001). Isotropic MAVRIC SL was more effective than reduced-TR MAVRIC SL (*p* < 0.032). ICC values 0.61–1.00
Kim et al. [74]	3T system MAVRIC SL STIR STIR	A cadaver 5 volunteers	Cadaveric study MAVRIC SL STIR better visualized anatomic structures, less distortion and pile-up. Fat suppression was better with STIR. Interobserver agreement κ = 0.7 Volunteer study MAVRIC SL STIR better visualized anatomic structures, less distortion. Spinal cord was better depicted by STIR. Interobserver agreement κ = 0.89

**Table 4:** Characteristics of modalities presented in the review.

	Advantages	Disadvantages
DT	The images are obtained with a single X-ray tube sweep, with a range of 8 to 40°, 25 to 76 projections. Images at arbitrary angles can be obtained. TMAR processing can improve image quality, in-depth spatial resolution, and lower radiation exposure compared to CT. Useful in evaluation of periprosthetic conditions after arthroplasty and bone formation after fractures or osteotomies.	DT cannot produce multiplanar reconstructions, as in CT, because number of projections and the acquisition angle are limited. Some new artifacts are generated, such as undershooting, blurring-ripple, and ghost artifacts.
CT	Using MBIR as the reconstruction algorithm reduces scatter and edge effects. MAR significantly reduces metal artifacts, particularly the effects of beam hardening and photon starvation. DECT can produce VMI which can be reconstructed at arbitrary average energy levels, optimize contrast, and reduce artifacts. • Currently, the combination of DECT and MAR is the best solution for metal artifact reduction.	Strong metal artifacts with conventional CT such as beam hardening, scattering, photon starvation, and edge effects. MAR may create new artifacts in the case of lightweight metals. Metal artifacts differ due to differences in the size, geometry, and alloys of metal implants.
MRI	Several MARS have been introduced: VAT, WARP, SEMAC, MAVRIC and MAVRIC SL. Each sequence has its own advantages. Currently, the combination of the CS acceleration technique with SEMAC may be the best solution in MRI to achieve metal artifact reductions with a reasonable acquisition time.	Traditionally, heavy metal artifacts, such as signal loss and distortion, lead to poor image quality. As imaging sequence becomes more complex, imaging time increases. Combined use of acceleration techniques is necessary to reduce imaging time.

## Data Availability

Not applicable to a review article.

## Abbreviations

Computed tomography: CT; digital tomosynthesis: DT; flat panel detectors: FPD; iterative reconstruction: IR; tomosynthesis with metal artifact reduction: TMAR; total hip arthroplasty: THA; metal artifact reduction algorithm: MAR; Computed tomography: **CT**; filtered back-projection: FBP; dual-energy CT: DECT; iterative reconstruction: IR; model-based iterative reconstruction: MBIR; metal artifact reduction algorithm: MAR; total hip arthroplasty: THA; total knee arthroplasty: TKA; virtual monoenergetic images: VMI; image optimization algorithms: IOA; differentiation algorithms: DA; quantification algorithms: QA; Magnetic resonance imaging: MRI; short inversion time inversion-recovery: STIR; signal-to-noise ratio: SNR; metal artifact reduction sequence: MARS; view angle tilting: VAT; slice encoding for metal artifact correction: SEMAC; multi-acquisition variable-resonance image combination: MAVRIC; MAVRIC selective: MAVRIC SL; negative predictive value: NPV; proton density weighted: PDW; Parallel imaging: PI; compressed sensing: CS.

## References

[1] Tang H., Yang D., Guo S., Tang J., Liu J., Wang D., Zhou Y. (2016). Digital tomosynthesis with metal artifact reduction for assessing cementless hip arthroplasty: A diagnostic cohort study of 48 patients. Skelet. Radiol..

[2] Koyama S., Aoyama T., Oda N., Yamauchi-Kawaura C. (2010). Radiation dose evaluation in tomosynthesis and C-arm cone-beam CT examinations with an anthropomorphic phantom. Med. Phys..

[3] Xia W., Yin X.-R., Wu J.-T., Wu H.-T. (2013). Comparative study of DTS and CT in the skeletal trauma imaging diagnosis evaluation and radiation dose. Eur. J. Radiol..

[4] Biswas D., E Bible J., Bohan M., Simpson A.K., Whang P.G., Grauer J.N. (2009). Radiation Exposure from Musculoskeletal Computerized Tomographic Scans. J. Bone Jt. Surg..

[5] Minoda Y., Yoshida T., Sugimoto K., Baba S., Ikebuchi M., Nakamura H. (2014). Detection of Small Periprosthetic Bone Defects after Total Knee Arthroplasty. J. Arthroplast..

[6] Solomon L.B., Stamenkov R.B., MacDonald A.J., Yaikwavong N., Neale S.D., Moss M.J., Howie D.W. (2012). Imaging Periprosthetic Osteolysis around Total Knee Arthroplasties Using a Human Cadaver Model. J. Arthroplast..

[7] Blum A., Noël A., Regent D., Villani N., Gillet R., Teixeira P.G. (2018). Tomosynthesis in musculoskeletal pathology. Diagn. Interv. Imaging.

[8] Nelson G., Wu M., Hinkel C., Krishna G., Funk T., Rosenberg J., Fahrig R. (2016). Improved targeting accuracy of lung tumor biopsies with scanning-beam digital X-ray tomosynthesis image guidance. Med. Phys..

[9] Tucker L., Gilbert F.J., Astley S.M., Dibden A., Seth A., Morel J., Bundred S., Litherland J., Klassen H., Lip G. (2017). Does Reader Performance with Digital Breast Tomosynthesis Vary according to Experience with Two-dimensional Mammography?. Radiology.

[10] McAdams H.P., Samei E., Dobbins J., Tourassi G., Ravin C.E. (2006). Recent Advances in Chest Radiography. Radiology.

[11] Dobbins J.T., Godfrey D.J. (2003). Digital X-ray tomosynthesis: Current state of the art and clinical potential. Phys. Med. Biol..

[12] Sakimoto T., Nishino K. (2013). Metal artifact reduction in tomosynthesis by metal extraction and ordered subset-expectation maximization (OS-EM) reconstruction. Medical Imaging 2013: Physics of Medical Imaging.

[13] Machida H., Yuhara T., Mori T., Ueno E., Moribe Y., Sabol J.M. (2010). Optimizing Parameters for Flat-Panel Detector Digital Tomosynthesis. RadioGraphics.

[14] Zotti M.G., Campbell D.G., Woodman R. (2012). Detection of Periprosthetic Osteolysis around Total Knee Arthroplasties: An in vitro study. J. Arthroplast..

[15] Machida H., Yuhara T., Tamura M., Ishikawa T., Tate E., Ueno E., Nye K., Sabol J.M. (2016). Whole-Body Clinical Applications of Digital Tomosynthesis. RadioGraphics.

[16] Dobres J., Chahine N., Reimer B. (2017). Effects of ambient illumination, contrast polarity, and letter size on text legibility under glance-like reading. Appl. Ergon..

[17] Ottenin M.-A., Jacquot A., Grospretre O., Noël A., Lecocq S., Louis M., Blum A. (2012). Evaluation of the Diagnostic Performance of Tomosynthesis in Fractures of the Wrist. Am. J. Roentgenol..

[18] Petraszko A., Siegal D., Flynn M., Rao S.D., Peterson E., van Holsbeeck M. (2016). The advantages of tomosynthesis for evaluating bisphosphonate-related atypical femur fractures compared to radiography. Skelet. Radiol..

[19] Geijer M., Gunnlaugsson E., Gotestrand S., Weber L., Geijer H. (2017). Tomosynthesis of the thoracic spine: Added value in diagnosing vertebral fractures in the elderly. Eur. Radiol..

[20] Tang H., Huang X., Cheng X., Yang D., Huang Y., Zhou Y. (2020). Evaluation of peri-prosthetic radiolucent lines surrounding the cementless femoral stem using digital tomosynthesis with metal artifact reduction: A cadaveric study in comparison with radiography and computed tomography. Quant. Imaging Med. Surg..

[21] Guo S., Tang H., Zhou Y., Huang Y., Shao H., Yang D. (2018). Accuracy of Digital Tomosynthesis with Metal Artifact Reduction for Detecting Osteointegration in Cementless Hip Arthroplasty. J. Arthroplast..

[22] Gillet R., Teixeira P., Bonarelli C., Coudane H., Sirveaux F., Louis M., Blum A. (2019). Comparison of radiographs, tomosynthesis and CT with metal artifact reduction for the detection of hip prosthetic loosening. Eur. Radiol..

[23] Toyooka S., Masuda H., Nishihara N., Shimazaki N., Ando S., Kawano H., Nakagawa T. (2020). Tomosynthesis Is Equivalent to Computed Tomography for Evaluating Osseous Integration after Anterior Cruciate Ligament Reconstruction. Arthrosc. Sports Med. Rehabil..

[24] Ishibashi K., Sasaki E., Wijaya E., Yamauchi S., Sasaki S., Kimura Y., Yamamoto Y., Shimbo T., Tamai K., Ishibashi Y. (2022). A Novel Quantitative Evaluation of Bone Formation after Opening Wedge High Tibial Osteotomy Using Tomosynthesis. J. Digit. Imaging.

[25] Okano E., Hara Y., Ito A., Mataki K., Totoki Y., Noguchi H., Nagashima K., Matsumoto Y., Yanagisawa Y., Mutsuzaki H. (2021). Novel method for selecting slices of the same cross-sectional view from digital tomosynthesis for monitoring posterior spinal instrumentation. J. Clin. Neurosci..

[26] Mataki K., Hara Y., Okano E., Nagashima K., Noguchi H., Shibao Y., Miura K., Takahashi H., Funayama T., Koda M. (2022). Development of a quantitative method to evaluate pedicle screw loosening after spinal instrumentation using digital tomosynthesis. BMC Musculoskelet. Disord..

[27] Gomi T., Sakai R., Hara H., Watanabe Y., Mizukami S. (2021). Usefulness of a Metal Artifact Reduction Algorithm in Digital Tomosynthesis Using a Combination of Hybrid Generative Adversarial Networks. Diagnostics.

[28] Gazaille R.E., Flynn M.J., Page W., Finley S., Van Holsbeeck M. (2011). Technical innovation: Digital tomosynthesis of the hip following intra-articular administration of contrast. Skelet. Radiol..

[29] Mallinson P.I., Coupal T.M., McLaughlin P.D., Nicolaou S., Munk P.L., Ouellette H.A. (2016). Dual-Energy CT for the Musculoskeletal System. Radiology.

[30] Boudabbous S., Arditi D., Paulin E., Syrogiannopoulou A., Becker C., Montet X. (2015). Model-Based Iterative Reconstruction (MBIR) for the Reduction of Metal Artifacts on CT. Am. J. Roentgenol..

[31] Wellenberg R., Hakvoort E., Slump C., Boomsma M., Maas M., Streekstra G. (2018). Metal artifact reduction techniques in musculoskeletal CT-imaging. Eur. J. Radiol..

[32] Choo H.J., Lee S.J., Kim D.W., Lee Y.J., Baek J.W., Han J.-Y., Heo Y.J. (2021). Comparison of the Quality of Various Polychromatic and Monochromatic Dual-Energy CT Images with or without a Metal Artifact Reduction Algorithm to Evaluate Total Knee Arthroplasty. Korean J. Radiol..

[33] Albrecht M.H., Vogl T.J., Martin S.S., Nance J.W., Duguay T.M., Wichmann J.L., De Cecco C.N., Varga-Szemes A., Van Assen M., Tesche C. (2019). Review of Clinical Applications for Virtual Monoenergetic Dual-Energy CT. Radiology.

[34] Nicolaou S., Liang T., Murphy D.T., Korzan J.R., Ouellette H., Munk P. (2012). Dual-Energy CT: A Promising New Technique for Assessment of the Musculoskeletal System. Am. J. Roentgenol..

[35] Barrett J.F., Keat N. (2004). Artifacts in CT: Recognition and Avoidance. Radiographics.

[36] Boomsma M.F., Warringa N., Edens M.A., Mueller D., Ettema H.B., Verheyen C.C.P.M., Maas M. (2016). Quantitative analysis of orthopedic metal artefact reduction in 64-slice computed tomography scans in large head metal-on-metal total hip replacement, a phantom study. Springerplus.

[37] Wellenberg R.H.H., Boomsma M.F., Osch van J.A.C., Milles J., Vlassenbroek A., Edens M.A., Streekstra G.J., Slump C.H., Maas M. (2016). Computed tomography imaging of a hip prosthesis using iterative model-based reconstruction and orthopaedic metal artefact reduction: A quantitative analysis. J. Comput. Assist. Tomogr..

[38] Wellenberg R.H.H., Boomsma M.F., van Osch J.A.C., Vlassenbroek A., Milles J., Edens M.A., Streekstra G.J., Slump C.H., Maas M. (2017). Low-dose CT imaging of a total hip arthroplasty phantom using model-based iterative reconstruction and orthopedic metal artifact reduction. Skelet. Radiol..

[39] Zhang K., Han Q., Xu X., Jiang H., Ma L., Zhang Y., Yang K., Chen B., Wang J. (2020). Metal artifact reduction of orthopedics metal artifact reduction algorithm in total hip and knee arthroplasty. Medicine.

[40] Hakvoort E., Wellenberg R., Streekstra G. (2020). Quantifying near metal visibility using dual energy computed tomography and iterative metal artifact reduction in a fracture phantom. Phys. Medica.

[41] Neuhaus V., Hokamp N.G., Zopfs D., Laukamp K., Lennartz S., Abdullayev N., Maintz D., Borggrefe J. (2019). Reducing artifacts from total hip replacements in dual layer detector CT: Combination of virtual monoenergetic images and orthopedic metal artifact reduction. Eur. J. Radiol..

[42] Bolstad K., Flatabo S., Aadnevik D., Dalenhaug I., Vetti N. (2018). Metal artifact reduction in CT, a phantom study: Subjective and objective evaluation of four commercial metal artifact reduction algorithms when used on three different orthopaedic metal implants. Acta Radiol..

[43] Barreto I., Pepin E., Davis I., Dean C., Massini T., Rees J., Olguin C., Quails N., Correa N., Rill L. (2020). Comparison of metal artifact reduction using single-energy CT and dual-energy CT with various metallic impants in cadavers. Eur. J. Radiol..

[44] Racine D., Ott J.G., Andreisek G., Omoumi P., Becce F., Verdun F.R. (2015). Dual-Energy CT: Basic Principles, Technical Approaches, and Applications in Musculoskeletal Imaging (Part 1). Semin. Musculoskelet. Radiol..

[45] Carotti M., Salaffi F., Beci G., Giovagnoni A. (2019). The application of dual-energy computed tomography in the diagnosis of musculoskeletal disorders: A review of current concepts and applications. La Radiol. Medica.

[46] Rajiah P., Sundaram M., Subhas N. (2019). Dual-Energy CT in Musculoskeletal Imaging: What Is the Role Beyond Gout?. Am. J. Roentgenol..

[47] Coupal T.M., Mallinson P.I., McLaughlin P., Nicolaou S., Munk P.L., Ouellette H. (2014). Peering through the glare: Using dual-energy CT to overcome the problem of metal artefacts in bone radiology. Skelet. Radiol..

[48] Lee Y.H., Park K.K., Song H.T., Kim S., Suh J.S. (2012). Metal artefact reduction in gemstone spectral imaging dual-energy CT with and without metal artefact reduction software. Eur. Radiol..

[49] Kuchenbecker S., Faby S., Sawall S., Lell M., Kachelrieß M. (2015). Dual energy CT: How well can pseudo-monochromatic imaging reduce metal artifacts?. Med. Phys..

[50] Horat L., Hamie M.Q., Huber F.A., Guggenberger R. (2019). Optimization of Monoenergetic Extrapolations in Dual-Energy CT for Metal Artifact Reduction in Different Body Regions and Orthopedic Implants. Acad. Radiol..

[51] Park C., Lee S.-M., Seo J.S., Kim T.W., Rhee S.J., Jeong H.S. (2022). Metal Artifact Reduction Dual-Energy CT as an Accurate and Reliable Method for Measuring Total Knee Arthroplasty Femoral Component Rotation Compared to Conventional CT. J. Knee Surg..

[52] Meinel F.G., Bischoff B., Zhang Q., Bamberg F., Reiser M.F., Johnson T.R. (2012). Metal Artifact Reduction by Dual-Energy Computed Tomography Using Energetic Extrapolation: A systematically optimized protocol. Investig. Radiol..

[53] Yoo H.J., Hong S.H., Chung B.M., Moon S.J., Choi J.-Y., Chae H.D., Chang M.-Y. (2018). Metal Artifact Reduction in Virtual Monoenergetic Spectral Dual-Energy CT of Patients with Metallic Orthopedic Implants in the Distal Radius. Am. J. Roentgenol..

[54] Lee K.Y.G., Cheng H.M.J., Chu C.Y., Tam C.W.A., Kan W.K. (2019). Metal artifact reduction by monoenergetic extrapolation of dual-energy CT in patients with metallic implants. J. Orthop. Surg..

[55] Donders J., Wellenberg R., Streekstra G., Maas M., Kloen P. (2020). Improved diagnostic confidence in evaluating bone non-union using virtual monochromatic dual-energy CT. Eur. J. Radiol..

[56] Andersson K.M., Nowik P., Persliden J., Thunberg P., Norrman E. (2015). Metal artefact reduction in CT imaging of hip prostheses-an evaluation of commercial techniques provided by four vendors. Br. J. Radiol..

[57] Bongers M.N., Schabel C., Thomas C., Raupach R., Notohamiprodjo M., Nikolaou K., Bamberg F. (2015). Comparison and combination of dual-energy and iterative-based metal artefact reduction on hip prosthesis and dental implants. PLoS ONE.

[58] Long Z., Delone D.R., Kotsenas A.L., Lehman V.T., Nagelschneider A.A., Michalak G.J., Fletcher J.G., McCollough C.H., Yu L. (2019). Clinical Assessment of Metal Artifact Reduction Methods in Dual-Energy CT Examinations of Instrumented Spines. Am. J. Roentgenol..

[59] Yue D., Rong C.F., Ning C., Liang H., Lian L.A., Xin W.R., Hong L.Y. (2017). Reduction of metal artifacts from unilateral hip arthroplasty on dual-energy CT with metal artifact reduction software. Acta Radiol..

[60] Chae H.-D., Hong S.H., Shin M., Choi J.-Y., Yoo H.J. (2020). Combined use of virtual monochromatic images and projection-based metal artifact reduction methods in evaluation of total knee arthroplasty. Eur. Radiol..

[61] Park H.S., Lee S.M., Kim H.P., Seo J.K., Chung Y.E. (2018). CT sinogram-consistency learning for metal-induced beam hardening correction. Med. Phys..

[62] Ronneberger O., Fischer P., Brox T., Navab N., Wells W.M., Hornegger J., Frangi A.F. (2015). U-Net: Convolutional network for biomedical image segmentation. International Conference on Medical Image Computing and Computer-Assisted Intervention, MICCAI 2015.

[63] Zhang Y., Yu H. (2018). Convolutional Neural Network Based Metal Artifact Reduction in X-ray Computed Tomography. IEEE Trans. Med. Imaging.

[64] Wang J., Zhao Y., Noble J.H., Dawant B.M. (2018). Conditional Generative Adversarial Networks for Metal Artifact Reduction in CT Images of the Ear. Med. Image Comput. Comput. Assist. Interv..

[65] Talbot B.S., Weinberg E.P. (2016). MR Imaging with Metal-suppression Sequences for Evaluation of Total Joint Arthroplasty. Radiographics.

[66] Olsen R.V., Munk P.L., Lee M.J., Janzen A.L., Xiang Q.S., Masri B. (2000). Metal artifact reduction sequence: Early clinical application. Radiographics.

[67] Fritz J., Meshram P., Stern S.E., Fritz B., Srikumaran U., McFarland E.G. (2022). Diagnostic Performance of Advanced Metal Artifact Reduction MRI for Periprosthetic Shoulder Infection. J. Bone Jt. Surg..

[68] Huang C., Chen Y., Ding H., Huang Z., Zhang C., Li W., Liu X., Tu Z., Zhang W., Fang X. (2022). Metal Artifact Reduction Sequences MRI: A Useful Reference for Preoperative Diagnosis and Debridement Planning of Periprosthetic Joint Infection. J. Clin. Med..

[69] Jungmann P.M., Ganter C., Schaeffeler C.J., Bauer J.S., Baum T., Meier R., Nittka M., Pohlig F., Rechl H., Von Eisenhart-Rothe R. (2015). View-Angle Tilting and Slice-Encoding Metal Artifact Correction for Artifact Reduction in MRI: Experimental Sequence Optimization for Orthopaedic Tumor Endoprostheses and Clinical Application. PLoS ONE.

[70] Lu W., Pauly K.B., Gold G.E., Pauly J.M., Hargreaves B.A. (2009). SEMAC: Slice encoding for metal artifact correction in MRI. Magn. Reson. Med..

[71] Hayter C.L., Koff M.F., Shah P., Koch K.M., Miller T.T., Potter H.G. (2011). MRI after Arthroplasty: Comparison of MAVRIC and Conventional Fast Spin-Echo Techniques. Am. J. Roentgenol..

[72] Kim J.K., Kim Y.J., Lee S., Yoon D., Lee R.W., Hong J.U., Ryu D.-S., Bae J. (2022). Metallic Artifact Reduction of Multiacquisition with Variable Resonance Image Combination Selective–Short Tau Inversion Recovery for Postoperative Cervical Spine with Artificial Disk Replacement: A Preliminary Study. J. Comput. Assist. Tomogr..

[73] Sutter R., Ulbrich E.J., Jellus V., Nittka M., Pfirrmann C.W.A. (2012). Reduction of Metal Artifacts in Patients with Total Hip Arthroplasty with Slice-encoding Metal Artifact Correction and View-Angle Tilting MR Imaging. Radiology.

[74] Sutter R., Hodek R., Fucentese S.F., Nittka M., Pfirrmann C.W.A. (2013). Total Knee Arthroplasty MRI Featuring Slice-Encoding for Metal Artifact Correction: Reduction of Artifacts for STIR and Proton Density–Weighted Sequences. Am. J. Roentgenol..

[75] Galley J., Sutter R., Stern C., Filli L., Rahm S., Pfirrmann C.W.A. (2020). Diagnosis of Periprosthetic Hip Joint Infection Using MRI with Metal Artifact Reduction at 1.5 T. Radiology.

[76] Takahashi T., Thaker S., Lettieri G., Redmond A., Backhouse M.R., Stone M., Pandit H., O’Connor P. (2022). Reliability of slice-encoding for metal artefact correction (SEMAC) MRI to identify prosthesis loosening in patients with painful total hip arthroplasty—A single centre, prospective, surgical validation study. Br. J. Radiol..

[77] Zochowski K.C., Miranda M.A., Cheung J., Argentieri E.C., Lin B., Kaushik S.S., Burge A.J., Potter H.G., Koff M.F. (2019). MRI of Hip Arthroplasties: Comparison of Isotropic Multiacquisition Variable-Resonance Image Combination Selective (MAVRIC SL) Acquisitions with a Conventional MAVRIC SL Acquisition. Am. J. Roentgenol..

[78] Fritz J., Guggenberger R., Del Grande F. (2021). Rapid Musculoskeletal MRI in 2021: Clinical Application of Advanced Accelerated Techniques. Am. J. Roentgenol..

[79] Khodarahmi I., Haroun R.R., Lee M., Fung G.S.K., Fuld M.K., Schon L.C., Fishman E.K., Fritz J. (2018). Metal artifact reduction computed tomography of arthroplasty implants: Effects of combined modeled iterative reconstruction and dual-energy virtual monoenergetic extrapolation at higher photon energies. Investig. Radiol..

[80] Khodarahmi I., Fritz J. (2017). Advanced MR Imaging after Total Hip Arthroplasty: The Clinical Impact. Semin. Musculoskelet. Radiol..

[81] Fritz J., Fritz B., Thawait G.K., Raithel E., Gilson W.D., Nittka M., Mont M.A. (2016). Advanced metal artifact reduction MRI of metal-on-metal hip resurfacing arthroplasty implants: Compressed sensing acceleration enables the time-neutral use of SEMAC. Skelet. Radiol..

[82] Jungmann P.M., Bensler S., Zingg P., Fritz B., Pfirrmann C.W., Sutter R. (2019). Improved Visualization of Juxtaprosthetic Tissue Using Metal Artifact Reduction Magnetic Resonance Imaging: Experimental and clinical optimization of compressed sensing SEMAC. Investig. Radiol..

[83] Filli L., Jungmann P.M., Zingg P.O., Rüdiger H.A., Galley J., Sutter R., Pfirrmann C.W.A. (2020). MRI with state-of-the-art metal artifact reduction after total hip arthroplasty: Periprosthetic findings in asymptomatic and symptomatic patients. Eur. Radiol..

[84] Fritz J., Ahlawat S., Demehri S., Thawait G.K., Raithel E., Gilson W.D., Nittka M. (2016). Compressed Sensing SEMAC: 8-fold Accelerated High Resolution Metal Artifact Reduction MRI of Cobalt-Chromium Knee Arthroplasty Implants. Investig. Radiol..

[85] Netto C.D.C., Schon L.C., Da Fonseca L.F., Chinanuvathana A., Stern S., Fritz J. (2019). Metal artifact reduction MRI for total ankle replacement sagittal balance evaluation. Foot Ankle Surg..

[86] Netto C.D.C., Fonseca L.F., Fritz B., Stern S.E., Raithel E., Nittka M., Schon L.C., Fritz J. (2018). Metal artifact reduction MRI of total ankle arthroplasty implants. Eur. Radiol..

[87] Khodarahmi I., Isaac A., Fishman E.K., Dalili D., Fritz J. (2019). Metal about the Hip and Artifact Reduction Techniques: From Basic Concepts to Advanced Imaging. Semin. Musculoskelet. Radiol..

[88] Christoph G., Falkowski A.L., von Deuster C., Nanz D., Sutter R. (2022). Basic and advanced metal-artifact reduction techniques at ultra-high field 7T magnetic resonance imaging-phantom study investigating feasibility and efficacy. Investig. Radiol..

[89] Kohyama S., Nishiura Y., Hara Y., Ogawa T., Ikumi A., Okano E., Totoki Y., Yamazaki M. (2021). A novel three-dimensional MRI-CT image fusion technique for precise preoperative evaluation and treatment of capitellar osteochondritis dissecans. Eur. Radiol..

[90] Kohyama S., Nishiura Y., Hara Y., Ogawa T., Ikumi A., Okano E., Totoki Y., Yoshii Y., Yamazaki M. (2021). Preoperative Evaluation and Surgical Simulation for Osteochondritis Dissecans of the Elbow Using Three-Dimensional MRI-CT Image Fusion Images. Diagnostics.

